# Screening of *Trypanosoma brucei gambiense* in Domestic Livestock and Tsetse Flies from an Insular Endemic Focus (Luba, Equatorial Guinea)

**DOI:** 10.1371/journal.pntd.0000704

**Published:** 2010-06-08

**Authors:** Carlos Cordon-Obras, Carmen García-Estébanez, Nicolás Ndong-Mabale, Simón Abaga, Pedro Ndongo-Asumu, Agustín Benito, Jorge Cano

**Affiliations:** 1 National Centre of Tropical Medicine (Institute of Health Carlos III), Madrid, Spain; 2 Reference Centre for Endemic Diseases Control in Equatorial Guinea, Ministry of Health and Social Welfare, Malabo, Equatorial Guinea; 3 National Sleeping Sickness Control Programme, Ministry of Health and Social Welfare, Bata, Equatorial Guinea; 4 National Programme for Malaria Control, Ministry of Health and Social Welfare, Malabo, Equatorial Guinea; Foundation for Innovative New Diagnostics (FIND), Switzerland

## Abstract

**Background:**

Sleeping sickness is spread over 36 Sub-Saharan African countries. In West and Central Africa, the disease is caused by *Trypanosoma brucei gambiense*, which produces a chronic clinical manifestation. The Luba focus (Bioko Island, Equatorial Guinea) has not reported autochthonous sleeping sickness cases since 1995, but given the complexity of the epidemiological cycle, the elimination of the parasite in the environment is difficult to categorically ensure.

**Methodology/Principal Findings:**

The aim of this work is to assess, by a molecular approach (Polymerase Chain Reaction, PCR), the possible permanence of *T. b. gambiense* in the vector (*Glossina* spp.) and domestic fauna in order to improve our understanding of the epidemiological situation of the disease in an isolated focus considered to be under control.

The results obtained show the absence of the parasite in peridomestic livestock but its presence, although at very low rate, in the vector. On the other hand, interesting entomological data highlight that an elevated concentration of tsetse flies was observed in two out of the ten villages considered to be in the focus.

**Conclusions:**

These findings demonstrate that even in conditions of apparent control, a complete parasite clearance is difficult to achieve. Further investigations must be focused on animal reservoirs which could allow the parasites to persist without leading to human cases. In Luba, where domestic livestock are scarcer than other foci in mainland Equatorial Guinea, the epidemiological significance of wild fauna should be assessed to establish their role in the maintenance of the infection.

## Author Summary

Sleeping sickness is a neglected disease with an important impact on public health of many countries of Sub-Saharan Africa. It is transmitted by tsetse fly bites (the vector) and mainly affects remote and rural populations. The chronic form, caused by *Trypanosoma brucei gambiense*, includes almost 90% of reported cases, and it is often misdiagnosed or lately detected after months or years of infection. Many efforts have been carried out to control the disease and interesting advances have been achieved. Although elimination is considered possible, there is an urgent need to understand the disease dynamic, especially in foci with very low rate (or absent) of infection for a long time. We performed a parasite screening in tsetse flies and livestock from Luba focus (Equatorial Guinea), considered to be “controlled” since 1995 (no human cases for fifteen years). The obtained results demonstrate that *T. b. gambiense* still remains in the environment and entomological data reveal high population density of the vector in some localities. This finding suggests that other intervention ways focused on control of vector populations, combined with the detection of human cases, could be necessary to achieve the total elimination of the parasite in hypoendemic foci.

## Introduction

Human African Trypanosomiasis (HAT), also known as sleeping sickness, is a parasitic disease endemic of the African continent. HAT is caused by two subspecies of the flagellate *Trypanosoma brucei*; *T. b. gambiense*, spread over West and Central Africa, which is responsible for the chronic form of the disease (more than 90% of total number of cases) and *T. b. rhodesiense*, which is present in East Africa and produces a few cases of acute infection per year. In addition, other members of *Trypanosoma* genus are able to infect a wide variety of animals producing diseases of veterinary importance such as nagana (*T. b. brucei*, *T. vivax* and *T. congolense*), surra (*T. evansi*) or dourine (*T. equiperdum*). *T. brucei s.l.* is mainly transmitted by tsetse flies (Diptera, Glossinidae) but other trypanosomes can be mechanically or sexually transmitted [1], [2].

In last years, control activities against sleeping sickness have been encouraged and significant advances were achieved to eliminate the disease [3]. The main strategy was to actively screen the human carriers in endemic foci [4], [5] since it is assumed that humans are the main reservoir of *T. b. gambiense* infection [6].

Luba focus, located on Bioko Island (Equatorial Guinea), is a good example of the success of control campaigns exclusively directed to humans. HAT was firstly declared in Luba in 1910 [7] and two decades later a successful control programme was implemented. At the end of 1960s, sleeping sickness was considered to be under control over the entire country and after the independence in 1968, HAT ceased to be a priority of public health for the new authority. In the middle of 1980s, Luba suffered a resurgence of the disease registering hundreds of cases leading to the establishment of the Sleeping Sickness National Control Programme (SSNCP) by Health Ministry and Social Welfare. This programme targeted the disease control combining active cases detection and passive surveillance using serological techniques. All parasitologically confirmed and serologically suspected cases were treated. This strategy led to a drastic reduction in the number of reported patients. The last autochthonous case was recorded in 1995 and no more surveys have been carried out since 2004 [5].

As occurred in Luba, the neglect of control activities in the past has led to a resurgence of HAT foci considered to have been eliminated. Several hypotheses could explain the resurgence of the disease in apparently controlled foci and the heterogeneity of the disease prevalence in neighbouring foci: movement of carrier populations from active foci [8], changes of the tsetse flies host preference [9], [10], genetic variability of the parasite [11], [12], the existence of asymptomatic parasite-infected individuals [13], inherent limitations of surveillance systems [14] and maintenance of infection in animal reservoirs. The latter theory is supported by the capability of the parasite for surviving in some species of domestic and wild animals [15]–[24].

This study aims to analyse *T. b. gambiense* infection in tsetse flies and domestic livestock from localities of Luba focus, in order to determine the presence of the parasites apart from the human transmission cycle. Species-specific molecular tools (PCR) were employed for diagnosis. In addition, entomological data about tsetse fly populations in these localities are provided and discussed.

## Materials and Methods

### Study Area

Luba focus covers a surface of 700 Km^2^ in south-western of Bioko Island. There are two climatic seasons: the dry season, from December to May, and the rainy season, from June to November. Bioko's annual rainfall exceeds 2,000 mm and the relative humidity ranges from 70% to 100% throughout the year. The average temperature is 25°C, with the minimum ranging from 17°C to 21°C and the maximum from 29°C to 30°C, depending on the location and the season [25].

The majority of the inhabitants from Luba lived on smallholding, hunting and sea fishing. Rainforest and neglected cocoa plantations are widespread in Luba district, establishing suitable habitats for the tsetse flies [26], [27]. Nowadays, many people from rural areas have migrated to urban, mainly to the capital city (Malabo), due to the recent development of petroleum and building industry. Therefore, rural conditions have partially disappeared and, as a result, many risk factors have been removed. Nevertheless, some villages remain unchanged and human-vector contact is still common.

### Sample Collection

In September 2007, blood samples of domestic animals (pigs, sheep and goats) were collected and tsetse flies were captured for further molecular analysis. Sampling procedures on Whatman filter paper for animal blood have been described elsewhere [28]. A previous census of livestock was elaborated in order to ensure a significant sample size. Ethical approval was obtained by Ministry of Health and Social Welfare and Veterinary Service from continental region (Ministry of Agriculture, Forestry and Environment). The study was conducted adhering to these institutions' guidelines for animal husbandry. Verbal informed consent was obtained from each owner of livestock prior to the extraction of blood samples by the field team.

**Figure 1:**
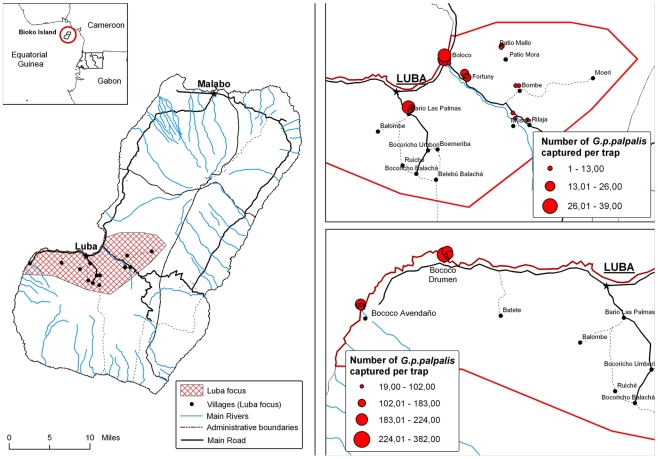
Map of Luba focus and distribution of tsetse fly captures over the villages.

Monopyramidal traps were employed to catch tsetse flies [29]. This kind of trap has been successfully applied for vector control and entomological surveillance in Equatorial Guinea [26], [30], [31]. This device makes flies to fall in a collecting bottle containing conservation solution (formaldehyde 5%) and to be stored until the gathering. Fifty-five traps were spread over the 10 villages belonging to epicentre of Luba focus. They were located in places considered *a priori* as suitable habitats for tsetse flies. This criterion includes water sources, cocoa or coffee plantations and shady and humid ponds close to livestock [32]–[34]. Geographical coordinates of all traps were registered by GPS (Figure 1) and they remained two weeks in the field. Sampling was carried out twice, a week after the placing and when the traps were removed.

Tsetse flies collected were stored in tubes with absolute ethanol in the field and separately processed in laboratory recording the trap number, an individual code, village, date, species, sex and age. The key of Brunhes *et al.* [35] was used for species identification and an age estimator, based on the degree of wear or fraying observed on the hind margin of the wing, was employed as previously described [36], [37]. In addition, apparent density (AD), estimated as AD = number of tsetse flies/trap/day, was calculated for each trap.

Both tsetse flies and blood samples were sent to National Centre of Tropical Medicine, Institute of Health Carlos III (Spain) for molecular processing.

### DNA Extraction and Molecular Analysis

DNA extraction from blood samples was performed employing a slightly modified protocol with Chelex 100® ionic resin (Bio-Rad Laboratories, Madrid, Spain) as previously described [28], [38].

Prior to DNA extraction, wings and legs of tsetse flies were removed using a sterile surgical blade. This step was carried out in order to minimize the amount of exoskeleton compounds included in the sample, which are known to inhibit subsequent enzymatic reactions [39]. Flies were then washed in 70% ethanol and in double distilled water (DDW). For dried samples, each fly was put in a sterile 1.5 ml tube and DNA extraction was performed employing the SpeedTools Tissue DNA Kit (Biotools, B & M Labs, S.A., Madrid, Spain) following the manufacturer instructions. All extraction instruments were sterilized after processing each fly by ethanol submersion and flaming. Finally, a negative control (clean 1.5 ml tube with no sample) was included in all procedures of the extraction (one negative each seventeen samples).

Ten µl of DNA template from blood samples were subjected to species-specific PCR for *T. brucei* s.l. and, when positive, for *T. b. gambiense*. For *T. brucei* s.l. analysis, TBR1/2 primers were used [40] with the following conditions: 1× PCR Reaction Buffer (10 mM Tris-HCl, 1.5 mM MgCl_2_, 50 mM KCl, pH 8.3), 200 µM of each deoxynucleotide (dNTP), primers at 0.5 µM and 1.25 U of *Taq* DNA Polymerase (Roche Diagnostics, S.L. Barcelona, Spain) in a final volume of 50 µl. Positive samples for this test were diagnosed for *T. b. gambiense* employing a nested-PCR with a first reaction using TgsGP1/2 primers [41] and a second one with TgsGP sense2/antisense2 primers described by Morrison *et al.* [42]. In both reactions 50 µl of final volume were reached and conditions were identical to *T. brucei* s.l. test with the exception of the amount of polymerase employed (2.5 U). The amplification programme for *T. brucei* s.l. was set as follows: a first step at 85°C (5 min) for hot starting, 3 min at 95°C for initial DNA denaturation, 40 cycles of 95°C (1 min), 55°C (1 min) and 72°C (1 min) and a final extension step at 72°C (5 min). For the first reaction of *T. b. gambiense* the fixed programme was: initial denaturation step at 95°C (5 min), 45 cycles of 94°C (1 min), 63°C (1 min) and 72°C (1 min) with a final extension step at 72°C for 5 min. The programme for the second reaction was identical but only 25 cycles were performed.

The quality of DNA templates from tsetse samples were tested by amplification of specific tubulin gene following the protocols described by Hao Z et al. (2003) [43] and Ferreira F et al. (2008) [44]. This step was considered because of the known PCR inhibition with samples of arthropods [39]. DNA samples that displayed a positive amplification signal for the tsetse tubulin gene were further tested to detect *T. brucei* s.l. and *T. b. gambiense* with the same primers and similar conditions as above: 1× PCR reaction buffer (10 mM Tris-HCl, 50 mM KCl, pH 8.3), 2 mM MgCl_2_, primers at 0.5 µM, 200 µM of each dNTP, 1 µl of DNA template and 1 U of AmpliTaq® Gold DNA Polymerase (Applied Biosystems, Branchburg, New Jersey, USA) reaching a final volume of 25 µl. The amplification programmes were modified increasing the time for the first denaturation step up to 10 minutes for polymerase activation as recommended by manufacturer and excluding the manual hot start step performed previously for *T. brucei* s.l. reactions.

In all PCR assays two negative controls (with DDW as template) and one positive control (1 ng DNA from *T. brucei* s.l. 328.114 or 1 ng from *T. b. gambiense* ELIANE) were included.

**Figure 2:**
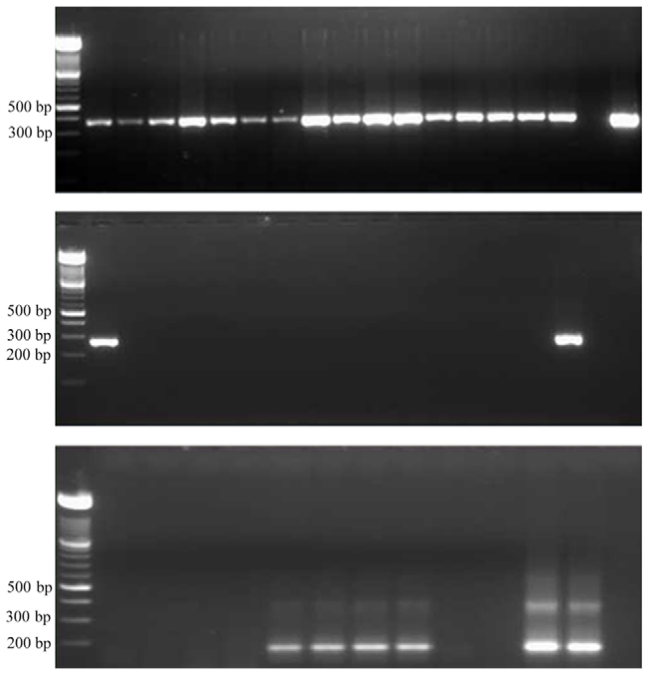
Revealed results of PCR in 2% agarose gel stained with ethidium bromide under UV. *Above:* Samples from sixteen tsetse flies submitted to PCR using GmTub primers as DNA quality control. Expected band size ∼380 bp. *Middle:* Molecular diagnosis of samples from fifteen tsetse flies for *T. b. gambiense*. First sample corresponds to the one positive tsetse fly. The band at right is the positive control. Expected band size 270 bp. *Below:* Detection of *T. brucei* s.l. in animal samples. 5–8 and 11 are positives, 12 is the positive control. Expected band size 177 bp.

All amplification products were separated by electrophoresis in a 2% agarose gel stained with ethidium bromide (1 µg/ml) and photographed under UV light. Any sample displaying a visible band of the expected length in the gel was considered as positive (Figure 2).

### Statistical Analysis

SPSS software (version 16.0.1, SPSS Inc., Chicago, IL, USA) was employed for statistical analysis and randomization of samples. Chi-square analysis was applied in order to compare significance of differences between variables (CI 95%). Given the low number of animals sampled in each village and subsequently positives for *T. brucei* s.l., no variables were statistically compared. Regarding tsetse flies, statistical associations between sex, age, sampling week, village and *T. brucei* infection were analysed, considering statistically significant p-value<0.05.

## Results

### Entomological Field Data

Overall 1,839 flies were collected. Almost all of them (1,830) were identified as *G. palpalis palpalis* as expected by previous entomological data in the country [5], [30], [31]. Eight flies were registered as *Glossina caliginea* and one individual could not be classified. Only two villages (Bococo Drumen and Bococo Avendaño) concentrated 89.7% of overall captures (1,650/1,839) (Table 1) and those localities showed a mean AD higher than 1 (14.9 in B. Drumen and 8.7 in B. Avendaño). The overall sex ratio was 1.59 (1,128 females/711 males) and no significant difference was observed between the sampling weeks (χ^2^ = 0.005, p = 0.942). However, sex ratio varies among the villages, from 1.01 in the lowest sampling localities to 1.33 in B. Avendaño and 2.2 in B. Drumen (χ^2^ = 19.2, p<0.001). Distribution of flies by age groups shows that during second sampling week collected individuals were significantly younger than in the first one (χ^2^ = 62.16, p<0.001).

**Table 1:** Distribution of tsetse fly captures and infection amongst villages.

Village	Total Captures 1^st^ week	Total Captures 2^nd^ week	Positives for *T. brucei* s.l./Total analysed	Positives for *T. b. gambiense*	AD*
B. Las Palmas	27	20	9/47	0/9	0.67
Fortuny (Boloco)**	57	27	20/81	0/20	0.88
Fortuny	22	17	7/38	0/7	
Musola	1	2	1/3	0/1	0.04
Rilaja	0	1	1/1	0/1	0.01
Bombe	1	1	1/2	0/1	0.03
Patio Mallo	9	5	3/12	0/3	0.2
B. Drumen	550	493	143/531	**1/143**	14.9
B. Avendaño	270	336	74/190	0/74	8.67
**Total**	**937**	**902**	**259/905**	**1/259**	**2.39**

No flies were trapped in Moeri or Patio Mora.

*AD = Apparent density calculated as number of flies/trap/day.

**Boloco belongs to Fortuny village but a set of five traps were located there due to its relative geographical distance.

### Molecular Analysis

A total of 951 tsetse flies gathered (761 randomly selected from B. Drumen and B. Avendaño and 190 flies collected from the other villages) were submitted to DNA extraction and specific tubulin amplification. From them, 905 (95.2%) yielded a positive result for tubulin amplification and then were considered to have enough DNA quality to be included in further diagnostic analysis. Overall, 28.6% (259) of these flies were positive for specific *T. brucei* s.l. PCR and only one (a young male fly from Bococo Drumen) showed a positive amplification for TgsGP being considered as carrier of *T. b. gambiense*. No significant difference in *T. brucei* s.l. prevalence was observed regarding the sex (χ^2^ = 0.708, p = 0.401), age (χ^2^ = 9.368, p = 0.095) or week of sampling (χ^2^ = 0.000, p = 1.00) but infection rate was significantly higher in B. Drumen than the others localities (χ^2^ = 42.43, p<0.001). Among the *G. caliginea* individuals (6) included in the analysis neither *T. brucei* s.l. nor *T. b. gambiense* were detected.

A previous census showed that there were 161 animals in villages belonging to the epicentre of Luba focus. Only 84 (52.2%) could be sampled since livestock were not kept in sheds and moved freely around the dwellings. Nine animals (eight pigs and one goat) (10.7%) yielded a positive result for *T. brucei* s.l. and none of them resulted positive for *T. b. gambiense* (Table 2).

**Table 2:** Distribution of animal sampling and infection amongst villages.

	Number of animals sampled/censed			Number of animals positive for *T. brucei* s.l.		
Village	Pigs	Goats	Total	Pigs	Goats	Total
B. Las Palmas	7/11	10/15	**17/26**	0/7	0/10	**0/17**
Fortuny (Boloco)	7/36	4/7	**11/43**	0/7	0/4	**0/11**
Fortuny	0/0	3/3	**3/6**	0/0	0/3	**0/3**
Patio Mallo	9/14	0/0	**9/14**	1/9	0/0	**1/9**
B. Drumen	24/40	4/4	**28/44**	4/24	1/4	**5/28**
B Avendaño	16/28	0/0	**16/28**	3/16	0/0	**3/16**
**Total**	**63/129**	**21/29**	**84/161**	**8/63**	**1/21**	**9/84**

Although three sheep were censed none of them could be sampled. In Musola, Rilaja, Bombe, Moeri and Patio Mora, no animals were present.

## Discussion

In order to investigate the presence of *Trypanosoma brucei gambiense* in Luba focus and its possible maintenance in a non-human transmission cycle, molecular diagnosis was performed on tsetse flies and peridomestic fauna. Our study has revealed the occurrence of one tsetse fly carrier of *T. b. gambiense*, demonstrating that parasite has not completely disappeared from the environment. Although only one individual was considered positive (prevalence ∼0.1%) this result is consistent with the typical low infection rate reached in the vector even in active foci [45], [46]. Regarding the domestic livestock no *T. b. gambiense* was detected, contrary to occur in other continental foci [28].

Prevalence of *T. brucei* s.l. was also determined in order to obtain an estimation of transmission activity in the focus. In tsetse flies, an overall high rate of infection was shown, although variations were observed regarding the village. A significantly higher tsetse flies infection rate was observed in Bococo Drumen from where it was gathered the majority of specimens collected in the focus during the study. In contrast, a relatively low prevalence of *T. brucei* s.l. in livestock was noticed, especially when compared with those previously described in continental foci [28]. Taking both data together it could be suggested that there is a high transmission activity, mainly in Bococo Drumen, but domestic fauna do not seem to act as the main feeding source for *G. p. palpalis* populations as in the other mainland foci.

A wild transmission cycle could explain all these epidemiological features: *G. p. palpalis* would mainly feed over wild fauna, which could lead to a high trypanosome infection rate of the vector and the maintenance of *T. b. gambiense* in the focus. Wild fauna would have a role as reservoir of *T. b. gambiense*, absent in peridomestic cycle, and it would be expected to show a higher prevalence for *T. brucei* s.l. than observed in livestock. In contrast with this situation, in Mbini focus (mainland Equatorial Guinea) domestic animals (sheeps and goats) have shown to be carriers of *T. b. gambiense* [28]. It should be pointed out that livestock breeding is more common in continental foci, where almost all villages have some kind of farming. In Mbini, around five hundred animals were censed in a previous study [28], whereas Luba only registered 161. A less availability of livestock could be other factor which favours the feeding preference of *G. p. palpalis* for wild fauna. Tsetse fly host preferences should be thoroughly studied in order to clarify this issue but the opportunistic feeding behaviour of this species described in several studies allows to hypothesize about a wild transmission cycle [47], [48]. Also, further research should be carried out in Luba to determine the role of wild animals in the maintenance of *T. b. gambiense* which has been previously described and discussed in neighbouring countries [15]–[17], [20], [22]–[24].

Some points about data must be added to this discussion. Firstly, the relative low sensitivity of the diagnostic technique employed for *T. b. gambiense* detection was previously reported since it targets TgsGP gene, only present once per haploid genome [49]. The low sensitivity of the test should not be *a priori* a serious drawback for tsetse flies samples since a great number of *Trypanozoon* subgenus parasites are usually found in infected midgut (around a maximum of 10^6^) [50] but it should be taken into account when data from animal blood samples are analysed. On the other hand, not all domestic animals were sampled and, as a result, it cannot be categorically ruled out the presence of the parasite in these hosts. These considerations should lead to weigh up the alternative hypothesis of the maintenance of infection in domestic livestock even in the absent of positive samples. In favour of this theory, it is noteworthy that Bococo Drumen and Bococo Avendaño, the villages where the tsetse fly density was higher, show the majority of domestic animals too. However, the low *T. brucei* s.l. infection rate found in livestock contradicts this apparent correlation. Future studies narrowly focused in feeding preferences of tsetse flies in Luba could clarify this issue.

Entomological data were also gathered in order to better understand the epidemiological dynamic of the focus. Surprisingly, it was shown that only two out of ten villages sampled in the epicentre of Luba exhibited a high density of vector population. Almost 90% of collected flies belonged to B. Drumen and B. Avendaño. These villages registered a significantly higher AD levels than the others which showed AD values according to the data observed in mainland foci [30], [31]. Factors such as rural condition, a more isolated location, a suitable environmental for resting and breeding (cocoa and coffee plantations) and the absence of vector control activities may have contributed to the spreading of tsetse fly populations in these villages. Moreover, the presence of wild and domestic animal hosts in this remote area of the southern Luba district could be the most conditioning factor in the vector densities. A higher sex ratio (female/male) of tsetse flies was also noticed in B. Drumen and B. Avendaño while this rate was near 50% in the other villages sampled. In previous studies carried out in mainland foci, a seasonal variation of sex ratio was observed, being higher after a peak of *G. p. palpalis* density typically reached at the end of rainy seasons [30], [31]. The sampling of this study was carried out during that period in order to collect as many flies as possible for a more accurate analysis and then, a high female/male ratio was expected. This pattern is more noticeable in the two villages with a higher density of vector where population seems to be well established and follow the natural dynamic. On the other hand, the individuals caught in the second week were younger, suggesting that the removal of flies during the first week had an impact over population dynamic. This phenomenon was also noticed in previous studies in this focus [26], [27]. It is consistent with the known low reproductive rate of *Glossina* genus and its relatively small population sizes [34], [51], [52].

Vector control was performed in Luba during a few months in order to assess its utility for reducing the human – vector contact. It was later given up and control activities were focused in active screening of human cases and passive detection in the district hospital [5], [27]. The high cost and maintained efforts needed for vector control strategies make it unsuitable in conditions of very low human infection rate. Nevertheless, strategies such as active screening, chemoprophylaxis with drugs, treatment of infection or spraying animals with insecticides to prevent bites of tsetse flies would not be viable in a wild cycle and hence, vector control would be the only option for an indirect intervention at this level. A vector control campaign focused in the areas surrounding villages with higher density of tsetse fly could reduce the vector - fauna contact, enabling a permanent elimination of the parasite in the epidemiological cycle.

In the last two decades, successful control campaigns have been carried out in Luba. Its insular situation gives a degree of isolation which makes more difficult the reintroduction of new cases or infected vectors from neighbouring countries, condition which allowed the tsetse fly elimination in others islands such as Zanzibar and Principe [53]–[55]. Other foci in the mainland Equatorial Guinea, where the same control activities were undertaken, have showed a fall of reported patients but *T. b. gambiense* infection was never completely cleared and a constant drop of cases per year is currently being described [3]. Other factors, such as economical changes (mainly petroleum exploitation) and the subsequent abandon of rural activities such livestock breeding and agriculture, could have contributed to the exceptional success of these control campaigns in Luba.

The results of this study suggest interesting features about trypanosomiasis epidemiology in Luba focus. Several differences have been noticed with regards to the other foci of Equatorial Guinea. *T. brucei* s.l. prevalence in domestic animals is much lower in Luba and no positive *T. b. gambiense* samples were found. By contrast, *T. brucei* s.l. infection rate in tsetse flies was high which could be a signal of an intense transmission. Taking into account both data, the hypothesis of the wild fauna as an important feeding source of *Glossina* spp. and *T. brucei* s.l reservoir should be considered. Although the prevalence rate is very low, *T. b. gambiense* infection in tsetse fly also confirms the theory of the permanence of this parasite in Luba focus. It could be concluded that controlling HAT in a given focus is a complex aim and different approaches must be addressed; conventional active human screening is an efficient strategy to decrease the number of cases but other interventions (such as vectorial control and management of other reservoirs) could be assessed in order to ensure the elimination of the parasite.

In the past, Luba suffered the effects of the neglect of successful control activities leading to a resurgence beginning the 1980s after more than 20 years of apparent absence of the parasite [26]. Nowadays, with the improved epidemiological knowledge achieved by decades of experience fighting the sleeping sickness, resurgences of this disease could be avoidable.
